# Oocyte Cryopreservation in Domestic Animals and Humans: Principles, Techniques and Updated Outcomes

**DOI:** 10.3390/ani11102949

**Published:** 2021-10-13

**Authors:** Theerawat Tharasanit, Paweena Thuwanut

**Affiliations:** 1 Department of Obstetrics, Gynecology and Reproduction, Faculty of Veterinary Science, Bangkok 10330, Thailand; 2 Veterinary Clinical Stem Cells and Bioengineering Research Unit, Chulalongkorn University, Bangkok 10330, Thailand; 3 Department of Obstetrics and Gynecology, Division of Reproductive Medicine, Faculty of Medicine, Chulalongkorn University, Bangkok 10330, Thailand; paweena.thuwanut@gmail.com

**Keywords:** animal, cryopreservation, human, oocyte

## Abstract

**Simple Summary:**

Oocyte cryopreservation is the most powerful technique for preserving the genetic potential of individual females. However, the recent outcomes of this technology in terms of viability, fertilizing ability, embryo development and pregnancy remain poor. The high sensitivity of the oocytes to freezing has been correlated with the profound dynamics of oocyte structures and functions. As a result, cryoinjury inevitably occurs at several cellular levels, which is indeed detrimental to cell viability and subsequent development. Advancement in the improvement of freezing technology via modifications to freezing technique and development of novel cryodevices plays a central role in mitigating cryoinjury and efficiently empowering the outcomes of oocyte cryopreservation. However, empirical study and optimizations of the techniques are generally required for cryopreservation of oocytes from particular species.

**Abstract:**

Oocyte cryopreservation plays important roles in basic research and the application of models for genetic preservation and in clinical situations. This technology provides long-term storage of gametes for genetic banking and subsequent use with other assisted reproductive technologies. Until recently, oocytes have remained the most difficult cell type to freeze, as the oocytes per se are large with limited surface area to cytoplasm ratio. They are also highly sensitive to damage during cryopreservation, and therefore the success rate of oocyte cryopreservation is generally poor when compared to noncryopreserved oocytes. Although advancement in oocyte cryopreservation has progressed rapidly for decades, the improvement of cryosurvival and clinical outcomes is still required. This review focuses on the principles, techniques, outcomes and prospects of oocyte cryopreservation in domestic animals and humans.

Assisted reproductive technologies, such as artificial insemination, in vitro fertilization and cryopreservation play important roles in basic research and the application of models for genetic preservation and in clinical situations. Of the technologies available, gamete preservation has increasingly gained attention because it provides long-term storage of gametes for genetic banking. The cryopreservation of somatic cells and sperm has been well adapted, and the success rate in terms of viability and functions is generally high. However, oocytes have limited surface area/cytoplasm ratio, which render them the most difficult cells to freeze. This review focuses on the principles, techniques, outcomes and prospects of oocyte cryopreservation in domestic animals, including small (dog and cat), medium-sized (pig) and large animals (cow and horse). Nonhuman primates and humans are also included in the review.

## 1. General Aspects of Oocyte Cryopreservation

The success of oocyte cryopreservation was first reported in the 1970s [1,2]. It has become clear that cryopreservation processes inevitably induce cellular and molecular changes that render poor fertilization rate and embryo development [3,4,5]. Controlled-rate slow cryopreservation and vitrification are the two freezing techniques that are clinically applied to oocyte cryopreservation in animals and humans. Slow freezing principally requires a relatively low concentration of cryoprotective agent (CPA), applied with sufficiently slow cooling/freezing rates to ensure a fine control over various factors (i.e., thermal shock) that contribute to cell damage [6]. By gradually decreasing the rate of supra- to sub-zero cooling, the CPA allows adequate cellular dehydration leading to minimal intracellular ice [6,7]. At subzero temperatures, the essential step of slow freezing is so-called “seeding”, a process which induces extracellular ice formation by converting the unfrozen solution to a hyperosmotic state, inducing cell dehydration [8]. With a slow-freezing approach, intracellular water is converted into a glassy phase composed of small intracellular ice crystals [7]. Therefore, super-rapid warming is required for the thawing process to avoid extensive crystallization and cell damage [7]. In contrast, vitrification requires an extremely high concentration of CPA and also an ultrafast freezing rate [9]. During cryopreservation, cells are exposed to several unfamiliar environments, such as chemical toxicity, osmotic changes and low temperature, all of which potentially disrupt cell functions and result in cell death [4,10,11]. Indeed, several factors, including species differences, age and fertility of oocyte donor, stage of oocyte maturation and cryopreservation protocols, have been reported to affect the success of oocyte cryopreservation. Notably, a large variation in oocyte physiology in particular animals cause difficulties in obtaining a consensus on freezing protocols. In some species, such as porcines, high contents of lipids have been claimed to cause poor oocyte freezing ability [12,13]. Thus, the development of freezing techniques and outcomes in terms of fertilization rate, embryo development and pregnancy rate following embryo transfer have been variable among species and laboratories. This aspect is very important for species for which oocytes are not readily available, such as wild species. In this case, anatomically and physiologically related domestic species are logically used to develop suitable cryopreservation techniques. Likewise, the availability of human oocytes for experimental purposes is very limited due to ethical reasons. According to similarities in reproductive physiology between nonhuman primates and humans, such as menstrual cycle length and hormonal profiles in rhesus macaques, nonhuman primates have been important in reproductive biology research during the last two decades [14]. Studies on nonhuman primates as human models in the fields of reproductive biology, reproductive medicine and assisted reproductive technology (ART) have been conducted for decades [14]. For oocyte cryopreservation, research studies using nonhuman primates were established in the late 1980s to early 1990s [15,16] where the principle of osmotic shock being responsible for oocyte quality was agreed [17]. In humans, the first achievement of pregnancy after oocyte cryopreservation was reported in 1986 [18]. However, limited success in oocyte cryopreservation discouraged this technique in routine clinical application for several years [19]. In the 2000s, knowledge of cryobiology by vitrification introduced the possibility of effectively cryopreserving functional oocytes, leading to revolutions in oocyte cryopreservation programs in clinical practice [20]. This was supported by a large randomized clinical trial on oocyte donation that revealed that vitrified oocyte quality was not inferior to fresh oocytes in terms of pregnancy outcomes [21]. Later, oocyte cryopreservation has become a fascinating alternative option for women who attempt in vitro fertilization (IVF) or fertility preservation programs [22]. Benefits of oocyte cryopreservation include increased flexibility to preserve (1) excess oocytes eventually present in each subsequent IVF cycle; (2) fertility in women who are at risk of infertility caused by chemotherapy/radiotherapy/premature ovarian insufficiency (POI) or who prefer to postpone childbearing and prevent age-related fertility decline (>36 years old) [20,22]. Additionally, oocyte cryopreservation technology could facilitate some advantages in routine IVF programs, such as (1) reducing the number of controlled ovarian stimulation cycles in infertile patients; (2) delaying fresh embryo transfer programs aimed at preventing ovarian hyperstimulation syndrome (OHSS) or to optimize artificial endometrial preparation (AEP) and (3) offering options for infertile couples with religious objections to embryo cryopreservation [5,23]. Consequently, oocyte cryopreservation is now considered a promising tool that could motivate women or infertile patients to preserve their genetic materials for medical or nonmedical reasons.

## 2. Principles of Oocyte Cryopreservation

Oocyte cryopreservation is an important tool for preserving germ cells for subsequent uses such as fertilization, as cytoplasts for somatic cell nuclear transfer, and also for genome banking for patients and valuable animal species. However, oocytes are very susceptible to damage during cooling and cryopreservation. Furthermore, oocytes also have a relatively low membrane permeability to water and cryoprotectants [24]. Although the optimization of freezing procedures has resulted in improvements in oocyte quality, oocyte structures such as the plasma membrane [25] and cytoskeleton [26,27] have been shown to be very sensitive to cryoinjury, frequently resulting in cellular disruption and cell death. Several factors have been shown to influence the outcome of oocyte cryopreservation, such as the stage of oocyte maturation during freezing, types of cryoprotectants used and freezing techniques. Immature oocytes are arrested at prophase I (germinal vesicle stage) where the condensed chromatins are protected within the nuclear membrane. Following maturation, the oocytes complete nuclear and cytoplasmic maturation promptly for fertilization and further embryonic development. Results obtained from the cryopreservation of immature and mature oocytes have been contradictory and variable among species and laboratories. In principle, the cryopreservation of immature oocytes is beneficial over mature oocytes, as they do not have a cold-sensitive meiotic spindle. However, cryopreservation processes per se disrupt oocyte structure and the signals responsible for oocyte maturation. Therefore, maturation and fertilization rates of frozen–thawed oocytes are generally poor when compared to noncryopreserved oocytes. These poor results of oocyte cryopreservation have been reported to involve cryoinjury at several levels, such as excessive formation of lethal intracellular ice [28], chromosome abnormality [29], disturbance of hyperosmotic stress [30,31], disruption of actins and microtubules [32] and zona pellucida hardening [33]. More recently, studies have also indicated that cryopreservation induces changes in gene and protein expressions [34,35,36,37].

## 3. Cryoprotective Agents

Cryoprotective agents (CPAs) are chemical substances generally used to protect from cryoinjury during cryopreservation. Notably, the actions of CPAs are variable according to the type used and other factors, such as temperatures and cell type [38]. The CPAs are broadly classified as penetrating and nonpenetrating CPAs according to their ability regarding cell membrane permeability.

Penetrating CPAs are generally organic compounds that permeate passively through the plasma cell membrane. These include glycerol, ethylene glycol, propylene glycol (1,2-propanediol), dimethyl sulfoxide, methanol and butanediol. Once the CPA enters the intracellular fluid, it replaces water and interferes in the hydrogen bonding between water molecules, thereby reducing intracellular ice formation. In addition, penetrating CPAs also help to increase the hydration status of cells during cooling in order to prevent the excessive accumulation of cellular electrolytes. Penetrating CPAs have, in general, a molecular weight typically less than 100 Daltons and with high amphiphilic properties [39]. However, excessive accumulation of penetrating CPAs also results in an increased cellular toxicity. Theoretically, intact mammalian cells should tolerate volume excursion, since these cells must be exposed to the hypertonic CPA solution [17,40]. This phenomenon leads to the difference in water activity between intra- and extra-cellular compartments [40]. If mammalian cells exceed the limitation of volume excursion at subzero temperatures, cell damage or apoptosis occur as a consequence of osmotic stress or osmotic shock [17,40]. It is worth noting that particular cell types will require a specific type and optimal concentration of CPA in order to protect them from cryoinjury during cooling and cryopreservation.

Nonpenetrating CPAs are another type of CPA commonly added to the freezing medium. These CPAs are high in molecular weight and often have high hydrophilicity. In past decades, researchers have applied nonpenetrating CPAs during cryopreservation in order to protect cells from high osmotic stress and CPA toxicity. The common nonpenetrating CPAs are polyvinylpyrrolidone, polyethylene glycol, and sugars such as sucrose and trehalose [41,42]. A combination of penetrating and nonpenetrating CPAs is the most practical way to reduce cryoinjury during cryopreservation [43,44,45]. Molecular dynamics of cell membranes (lipid bilayers) stimulated by CPAs have been regulated in various mechanisms which are dependent upon the types of CPAs. For example, dimethyl sulfoxide has a more efficient capability to diffuse across the phospholipid bilayers compared with the other CPAs. However, high concentrations of dimethyl sulfoxide might cause the thinning of lipid bilayers, thereby leading to the complete destruction of lipid bilayers or the loss of membrane permeability [38]. In contrast to the polyol CPAs (i.e., sugar alcohol), their ability to form hydrogen bonds with lipid bilayers could minimize the lipid bilayers-thinning effect [38]. Furthermore, another proposed mechanism is such as colligative property (alteration: phase of diagram in solution) which has been observed in glycerol. This mechanism could buffer salt (NaCl) concentration and alter crystalline solid forming occurred during cryopreservation process [38].

## 4. Cryopreservation Techniques

Techniques for the cryopreservation of oocytes, as well as of sperm and embryos, are generally classified as controlled-rate slow freezing and “ice-free” vitrification. Conventional slow freezing requires a programable freezer that can substantially control the optimal freezing rate. During cooling, the temperature is gradually decreased to below the freezing point where ice is formed. However, ice formation occurs in the extracellular and intracellular regions. Excessive ice formation within cells, especially intracellular ice formation, disrupts cell structure and function, which results in apoptosis or cell death. The initiation of the outgrowth of extracellular ice formation via seeding ice crystals is generally performed to mitigate the excessive formation of ice during supercooling. At this stage, extracellular ice is formed and the osmolarity of the extracellular fluid is also gradually increased. The oocytes will be in the dehydrated stage during freezing due to the unfrozen intracellular water flowing out to balance the osmolarity. As the oocytes are the largest cells and have low membrane permeability to cryoprotectants, most cryopreservation requires a freezing rate that is slow enough for sufficient CPA permeability. However, oocyte membrane permeabilities to CPA and cryotolerant have been demonstrated to differ among species. Although theoretical models can be used to predict the optimal freezing rate, empirical study is frequently required to test the freezing protocols prior to use. If the temperature is reduced too rapidly, excessive intracellular ice will be formed. In contrast, oocytes will undergo severe dehydration if the freezing rate is too slow. Therefore, the optimal freezing rate is the slow process that achieves a balance between adequate cellular dehydration and minimal intracellular ice formation. By using this technique, low concentrations of CPA are generally required, thus minimizing osmotic shock and CPA toxicity.

In contrast to slow freezing, vitrification allows the rapid transition from a liquid phase to a glasslike stage or water solidification. Vitrification is another promising technique for living cell cryopreservation [19]. Principally, the definition of vitrification is a “process of glass solidification of a liquid or water-based solution without ice crystal formation” [46]. To achieve this result, high concentrations of CPAs (both permeable and nonpermeable CPAs) are loaded onto living cells before deep freezing in liquid nitrogen [19,47]. However, this procedure causes extreme osmotic stresses and chemical toxicity [19,47]. Different devices can be modified for efficient vitrification such as open-pulled straw [48], solid surface [3], cryoloop [49], electron microgrids [50] and cryotop [51].

## 5. Outcome following Oocyte Cryopreservation

Oocyte cryopreservation is a mostly successful procedure in laboratory animals, especially in the mouse, due to the fact that the oocytes are quite tolerant to cold stress [52]. Additionally, the technology of embryo production from frozen oocytes is well developed in this species [53]. In the mouse, oocyte cryopreservation is very useful technique for storing the genetics of specific breeds such as gene-modified animals. Unlike mouse oocytes, knowledge on mechanism of oocyte maturation in other animals is still lacking. Although the oocytes of larger species are relatively more sensitive to cold stress compared to those of the mouse, live offspring have been born from medium to large size domestic animals, including cat [54,55], pig [56], cows [57,58,59] and horse [60,61], as well as humans [18].

### 5.1. Ruminants (Bovine and Ovine)

In ruminants, oocyte cryopreservation was first reported in the 1900s, indicating the possibility of embryo development and pregnancy following oocyte cryopreservation [57,62]. The previous findings also suggested that the mature oocytes were highly sensitive to cooling to lower than 10 °C, even without cryopreservation [63]. However, both stages of maturation (immature and mature stages) could be slow-cryopreserved with no significant difference in terms of cleavage and blastocyte formation rates (58% vs. 60% and 7% vs. 12%, respectively) [64]. The high chilling sensitivity of matured bovine oocytes led to the development of vitrification using electron microgrids [50]. In recent years, vitrification of bovine oocytes has gained interest. Indeed, full-term development following transfer of embryos derived from frozen–thawed or vitrified immature oocytes has been reported [58,59,64,65]. Development of vitrification technology for bovine oocytes has made much progress, probably because vitrification requires less equipment, is less time-consuming and is much more cost-effective compared to conventional slow freezing. Several devices have been applied efficiently in the vitrification of bovine oocytes, such as cryoloops [66], open-pulled straws [48] metal solid surfaces [3], cryotops [67], nylon mesh [68] and silk fibroin sheets [69]. The improvement in fertilization and blastocyst rates of vitrified oocytes has attributed to the extremely high cooling and warming rates and the use of a minimum-volume approach [48,70,71]. The latter technique, The Cryotop^®^ system, requires less than 0.1 µL volume and can reach a cooling of up to 23,000 °C/min and a warming at up to 42,000 °C/min. This system yields cleavage rates of 59.5% and blastocyst rates of 22.9%. However, this is still lower than fresh control (77.6% and 44.7%, respectively) [71]. Using minimum-volume vitrification, silk fibroin, nylon mesh and cryotop yielded similar blastocyst rates (approximately 25%) comparing unfavorably to controls (40.6%) [69]. Information regarding cryopreservation in sheep and goat is relatively limited compared to that in cows. While the outcome in terms of embryo development following conventional slow freezing is poor [72,73], most research has adapted the vitrification technologies from other species aimed at reducing cryoinjuries [72,74,75,76,77,78,79,80]. However, blastocyst development is still poor, ranging from 0% to 12.5% [75,76,77,81].

### 5.2. Horse

Research into the development of oocyte cryopreservation of equine oocytes has been limited, due principally to the difficulty in obtaining oocytes from equine ovaries. Equine ovaries can be obtained from local abattoirs in some countries or from live donors using ovum pickup (OPU) [82,83]. However, the relatively poor responses of equine ovaries to routine gonadotropin preparation [84] and also the poor oocyte recovery following OPU are due to the finding that equine cumulus cells adhere strongly to the follicular wall [85]. More importantly, conventional in vitro fertilization failed to fertilize the matured oocytes, and intracytoplasmic sperm injection (ICSI) is the only meaningful way of fertilizing the oocytes [86]. Variable results in meiotic competence and embryo development for cryopreserved equine oocytes are affected by several factors, such as the stage of maturation and the freezing medium and technique used. Information on the efficiency of slow freezing for equine oocytes is limited. Comparing the two techniques, open-pulled straw vitrification of immature oocytes yielded significantly greater maturation rates than slow freezing [32]. In addition, cryopreservation also induced alterative changes in mitochondrial morphology [87], cytoskeleton and chromatin configuration [32,88]. In the early phase of technological development, maturation rates of vitrified immature oocytes were poor (less than 20%) [87,89]. Recently, results have been much improved and range from 33.3% to 54% [61,90,91,92,93]. Following fertilization via ICSI, the vitrified–warmed oocytes could develop into cleavage- and blastocyst-stage embryos, but the efficiency is still poor when compared to nonfrozen oocytes [92,94]. First reports of pregnancy were obtained by the transfer of vitrified matured oocytes into the oviducts of inseminated mares [60]. More recently, pregnancy from vitrified oocytes and in vitro embryo culture could be obtained, with pregnancy rates of 17–50% and resulting in live foals [61,93].

### 5.3. Pig

In pigs, the development of cryopreservation of oocytes has been limited, due principally to the high sensitivity of porcine oocytes to cold stress [56,95]. Although the reason for this is entirely unclear, intracellular lipid content within porcine immature oocytes has been shown to be 2.4-fold higher than in bovine oocytes [96]. Although the removal of cytoplasmic lipids is detrimental to embryo development [97], partial delipidation of porcine oocytes prior to vitrification improved freezing tolerance and embryo development [12,98]. Unlike other species, the use of slow freezing has been unsuccessful in terms of survival and embryo development [99]. Because porcine oocytes are very sensitive to cryopreservation, most studies on porcine oocyte cryopreservation used vitrification. Since the first piglets were born after vitrification [56], the efficiency of this technique was much improved in the late 2000s. Modifications of vitrification devices, the type of cryoprotectant and the procedure used were tested, aiming at reducing cryoinjuries and cellular changes of the vitrified–warmed oocytes. Notably, vitrification induces several changes within porcine oocytes, such as in the cytoskeleton [100], mitochondrial abnormalities [101], epigenetic changes [102] and decreased expression level of the Type 1 inositol 1,4,5 trisphosphate receptor [103]. More recently, vitrification of porcine oocytes has been associated with alterations at transcriptomic and proteomic levels [37,104]. Similar to other species, both immature- and mature-stage oocytes have been successfully cryopreserved, either by conventional slow freezing or vitrification [105]. In direct comparison, vitrification of immature oocytes results in better cell cytoskeleton rearrangement [106] and embryo development [107]. Additionally, vitrification has been shown to induce parthenogenic activation at a high rate (approximately 50%) when oocytes are vitrified at the mature stage [108]. Disruption of cellular functions, especially mitochondrial activities, after vitrification leads to increased reactive oxygen species levels and results in cell apoptosis [109,110,111]. Therefore, the application of antioxidants such as astaxanthin [112] and caspase inhibitor [113] decreased apoptosis and improved the development of vitrified–warmed oocytes.

### 5.4. Canines and Felines

The cryopreservation of canine oocytes has been ignored due to the fact that canine oocytes are poorly resumed to reach metaphase II under in vitro culture conditions. Unlike other species, canine oocytes ovulate at an immature stage. The resumption of meiosis then takes place within the oviduct [114,115]. In most cases, matured oocytes would need to be collected from the oviducts, as the immature oocytes require 48–72 h of maturation time [115]. Clinically, the stage of oocyte maturation is predicted according to ovulation time or progesterone levels [116,117]. In in vitro conditions, small numbers of oocytes resumed meiosis and only 15% reached metaphase II stage. Although puppies have been born using IVF techniques, in vitro embryo production systems in this species have yet to be developed. Only two publications have reported attempts to vitrify canine oocytes [118,119] and only 3.9% of vitrified–warmed oocytes reached MII stage, comparing unfavorably to 8.2% MII of nonvitrified control oocytes [118]. The oocytes of nondomestic canines (the endangered Mexican gray wolf, *Canis lupus baileyi* and blue fox, *Alopex lagopus*) were also vitrified in order to use this technology for species conservation [120,121]. At the time of this report, no data on embryo development or pregnancy resulting from cryopreserved domestic or nondomestic canine oocytes are available.

In comparison with canines, the development of cryopreservation technology in domestic cats has progressed, probably due to oocyte availability and also the well-established in vitro embryo production system [122,123,124,125,126,127]. Until recently, the development of frozen oocytes in terms of blastocyst development and pregnancy rate has been poor and limited. Full-term development of kittens from vitrified matured oocytes has been reported [54,55]. Pregnancy could also be obtained from vitrified immature oocytes, but reabsorption unfortunately occurred [45]. Until recently, no full-term development of cryopreserved immature oocytes has been reported. It is hypothesized that feline immature oocytes are very sensitive to cryopreservation and also have limited tolerance to aniosmotic conditions [128]. The effects of the meiotic stage on the freezing ability of feline oocytes are still controversial. Indeed, feline oocytes have been successfully cryopreserved using conventional freezing and vitrification at both meiotic stages. Using conventional slow freezing, MII oocytes are significantly more tolerant to slow freezing compared with immature oocytes when cryopreserved slowly with ethylene glycol (cleavage rate 6.8% vs. 38.7%) [129]. Similarly, the cleavage rate of vitrified–warmed feline mature oocytes is slightly higher than that of the immature counterparts (21% vs. 4%) [130]. Development of the cryopreservation of feline oocytes focused on the use of vitrification yielded 0–10% blastocyst rates [54,55,131,132,133,134]. Modifications of the vitrification technique for immature oocytes, such as stepwise ethylene glycol exposure and inhibition of apoptosis with Rock inhibitor improved blastocyst rates (30.2% and 12.1%, respectively) [45,135].

### 5.5. Nonhuman Primates

In 1996, a study on the cynomolgus monkey or crab-eating macaque (*Macaca fascicularis*) demonstrated that the F-actin microfilament system in oocytes was modified by glycerol exposure at an ambient temperature [136]. Furthermore, abnormal morphology, represented by irregular shrinkage of oocytes, was observed in germinal vesicle (GV) and metaphase I oocytes after equilibration (room temperature to 0 °C) under different concentrations of glycerol (1.0–2.0 M) [136]. For the rhesus monkey (*Macaca mulatta*), a study revealed that dimethyl sulfoxide and ethylene glycol could effectively diffuse through the oocyte cell membrane. However, the glycerol was less permeated [17]. Additionally, oocyte (immature and mature) membrane integrity was abrupt when exposed to high concentrations of CPA (from 0.1 to 5 mol/L) [17]. A later study in 2005 revealed that rhesus macaque immature oocytes were less susceptible than mature oocytes to injury resulting from a hyperosmotic solution of ethylene glycol during the equilibration phase [137]. Immature oocytes cryopreserved by rapid nonequilibrium cooling suffered less damage compared to slow equilibrium cooling [138]. This was evidenced by the integrity of the microtubules and the intactness of transzonal processes between cumulus cells and oocytes in the rapid-freezing group [138]. Although research studies related to oocyte cryopreservation in nonhuman primates have not in progressed over the last 10 years, nonhuman primate research models were primary keys in understanding the factors involved in oocyte osmotic susceptibility, which could potentially provide a relevant platform for oocyte cryopreservation in humans [14].

### 5.6. Humans

The technology for oocyte cryopreservation in humans is well developed compared with other domestic species because this technology is highly important for clinical prospects. Therefore, chronological development and clinical outcomes are additionally highlighted in this review. The first successful pregnancy outcome using cryopreserved human oocytes was reported in 1986 [18]. The retrieved oocytes were slow-frozen with 1.5 mol/L dimethyl sulfoxide. However, a limited number of successful pregnancies by oocyte cryopreservation and conventional IVF have been reported. The growing interest in oocyte cryopreservation has been renewed due to the introduction of ICSI technology in the 1990s [5]. This technique could overcome zona pellucida hardening caused by the cryopreservation process, dramatically increasing the clinical pregnancy rate in IVF programs [5]. Since then, several efforts have been made to optimize oocyte cryopreservation protocols, both slow freezing and vitrification, with the ultimate goal of improving pregnancy outcomes and live birth rate [5,18]. After the achievements in embryo cryopreservation and ICSI technologies, a renaissance in oocyte cryopreservation was launched in the early 1990s [8]. Human oocytes present a low surface area to volume ratio, resulting in a high susceptibility to intracellular ice formation [139]. Comparative studies in mice and humans during the early 1990s highlighted difficulties in maintaining membrane permeability and the integrity of human oocytes during hyperosmotic solution exposure [140]. Thus, promising freezing protocols were then developed. In 1993, slow freezing using permeable and nonpermeable CPAs (1,2-propanediol (PROH) and sucrose) was performed on mature (MII) oocytes [141]. By this protocol, oocyte survival rate reached 64% after thawing, and normal spindle and chromosome configurations were observed in 60% of viable oocytes [141]. Furthermore, cryopreserved oocytes that had undergone normal fertilization achieved development of all two sets of 23 chromosomes [142]. Until the late 1990s to early 2000s, promising cryopreservation protocols progressed aimed at obtaining higher post-thaw survival. Notable results indicated that survival, implantation and pregnancy rates from cryopreserved oocytes were similar to those from post-thawed embryos [19,143,144]. These implications revoked research protocols applied on a routine basis and commercial freezing media kits [19,143,144]

Another interesting aspect besides oocyte survival rate and quality is genetic alteration during the cryopreservation process. One study in 2012 indicated that slow freezing was associated with the downregulation of genes involved in chromosomal structure maintenance (Kinesin-like protein; KIF2C and KIF3A) and cell cycle regulation (Checkpoint Kinase 2, CHEK2; and Cyclin Dependent Kinase Inhibitor 1B, CDKN1B) that possibly affected oocyte developmental competence [145]. Although pregnancy outcomes are accomplished by oocyte cryopreservation using the slow freezing method, limited numbers of novel research studies have been reported during the last decade. The primary obstacles were the high cost of a programmable freezer and the time-consuming nature of the procedure. Thus, an alternative strategy that could positively influence oocyte survival outcomes and be comparable to the slow freezing technique without using costly equipment was introduced. For human MII oocytes, vitrification was first applied to long-term preservation in 1989 using high concentrations of permeable (DMSO) and nonpermeable (sucrose) CPAs with ultrarapid freezing and thawing rates [146]. Promising results were observed, with more than 80% intact oocyte morphology [146]. Similar to slow freezing, this cryopreservation procedure was in the development phase for several years [19,146]. The first successes in pregnancy outcomes and live births using vitrified oocytes were reported in 1999 [147]. A total of 11 out of of 17 vitrified oocytes (64.7%), using 40% EG and 0.6 mol/L sucrose, survived. Pronuclear formation was observed in five of them after ICSI. A euploid embryo was transferred to an infertile patient with the final success of a live birth [147]. The initial protocols for oocyte vitrification were emphasized for cryosurvival [148]. The key to successful oocyte survival after vitrification were optimal cooling and warming rates [148,149]. In the meantime, another report minimized vitrification solution volume (a droplet of <0.1 μL compared to other protocols using 0.1–0.5 μL) and used a well-designed cryodevice that led to ultrarapid freezing and thawing (40,000 °C/min) rates [71]. This technique markedly yielded 100% morphologically intact oocytes and 52% blastocyst rates [71]. In particular, limited knowledge related to the detrimental effects of CPA toxicity and the vitrification procedure was investigated at the intracellular level [150]. During the early 2000s, simple low-resolution morphological assessment was used, which was not fully adequate to evaluate oocyte quality, fertilization potential or developmental competence [150]. Thus, intracellular oocyte-organelle studies were introduced using several techniques and types of equipment, i.e., polarized light microscopy (PLM), transmission electron microscopy (TEM) and epifluorescence or confocal laser scanning microscopy (CLSM) [150]. These technologies potentially provided great detail on the cryoinjuries in each individual vitrified oocyte [150]. For example, TEM micrography could demonstrate vacuolization present in cryopreserved oocyte ooplasm [150]. A lower degree of vacuolization in vitrified oocytes was observed compared with those from a slow freezing procedure [151]. For other organelles, mitochondrial–smooth endoplasmic reticulum (M–SER) aggregation observed by TEM was present in both vitrified–warmed or slow-frozen oocytes [152,153]. Supporting data indicated that an equilibration and freezing solution containing low EG concentration could decrease M–SER aggregation events [153]. The appearance of vacuoles or M–SER aggregation in human MII oocytes can possibly reduce oocyte fertilization and impair embryo development competence by disturbing Ca^2+^ homeostasis [150].

Not only morphological or ultrastructural oocyte damage but also patterns of genetic change should be of concern during the cryopreservation process. In the genome era, the impact of oocyte vitrification on genetic and epigenetic changes has been comprehensively studied during the last 10 years. For instance, the first study of gene alteration in vitrified human oocytes was published in 2010 [154]. Expression profiles of messenger RNA (mRNA) in single vitrified–thawed oocytes were compared with fresh cohorts [154]. Data revealed that genes encoding proteins essential for oocyte development and specific functions (bone morphogenetic protein 15 (BMP15), growth differentiation factor 9 (GDF9), folliculogenesis-specific basic helix-loop-helix (FIGLA), POU Class 5 homeobox 1 (POU5f1-OCT4) and TATA box binding protein-associated factor 4B (AF4B)) were not altered by vitrification using a commercial vitrification kit [154]. The number of genes studied in 2021 was between fewer than 10 and almost 2000 [155]. Differentially expressed genes were found in vitrified–thawed oocytes compared to fresh MII oocytes (1646 and 341 genes were downregulated and upregulated, respectively) [155]. In cryopreserved oocytes, genes related to oxidative phosphorylation, the lysosome pathway, regulation of lipolysis in adipocytes or the AMPK signaling pathway were upregulated [155]. On the epigenome issue, a recent study in sibling donor oocytes (fresh and vitrified samples) revealed that global DNA methylation (DNA (hydroxy)methylation pattern) did not differ between cohorts [156]. Furthermore, no significant differences were seen in cleavage timing or predictive morphokinetic time intervals between embryos developed from fresh and vitrified oocytes evaluated under time-lapse monitoring [156]. Another point of epigenetic issue, noncoding RNA (miRNA) expression in fresh/vitrified human oocytes was reported in 2019 [157]. At least 22 miRNAs differed between fresh and vitrified oocytes, e.g., miR-134-5p, miR-210-5p, miR-21-3p and miR-465c-5p, which target the PTEN gene (cell apoptosis regulation through oxidative stress pathway) [157]. However, a small sample size was used in these novel genome reports, and correlation between gene expression and other oocyte quality parameters were rarely determined [155]. Hence, other genetic and epigenetic alterations in human oocyte cryopreservation should be further elucidated.

## 6. Prospects and Conclusions Regarding Oocyte Cryopreservation

Advancement in oocyte cryopreservation has progressed rapidly for decades with satisfactory outcomes, but the study of cryobiology aimed at improving outcomes for the cryopreserved oocyte is still challenging. However, development and success rates in terms of survival and developmental competence are generally poor when compared to noncryopreserved oocytes. Additionally, the degree of oocyte susceptibility to cold stress and cryodamage is highly dependent upon species and the freezing technology used. Animal models are used to investigate the principle and effectiveness of freezing protocols, which later can be used efficiently with other reproductive technologies. The purposes of oocyte cryopreservation are globally similar between animals and humans; however, the ultimate aim in animals is to preserve oocytes for subsequent use and for “long-term” genome resource banking in wild species [158]. In humans, oocyte cryopreservation is clinically adapted as a tool for preserving fertility in cases of its premature loss, such as in women who need gonadotoxic chemotherapy for cancer treatment [159,160,161,162]. Indeed, oocytes differ from other cell types such as somatic cells and sperm cells as they are larger, have a low surface area to volume ratio, and also low membrane permeability to water and CPA, all of which make oocytes susceptible to damage during cryopreservation [24]. As a result, several types of cryoinjury, such as disorganization of microtubules and chromatin configuration, zona pellucida hardening, apoptosis and genetic and epigenetic alterations will inevitably occur when cryopreservation is carried out. It becomes clear that the cryopreservation of oocytes in animals remains at the experimental stage, as the outcomes in terms of live offspring are very limited. Of course, the ultimate goal in the study of oocyte cryopreservation would be to determine the factors that influence cryoinjury and to minimize their effects on the biological functions of the cryopreserved oocytes. Indeed, empirical studies on animals and variations in the protocols used have led to difficulties in comparing results among laboratories. However, the development of freezing techniques, in particular vitrification, is the most noticeable revolution in cryopreservation. This is likely due to the fact that vitrification can avoid the lethal ice formation seen in conventional slow freezing [19]. In addition, vitrification is less time-consuming and more cost-effective. In experimental phases, most researchers attempt to modify cryodevices to increase cooling and warming rates and also to modify different types of CPAs. However, the variations in oocyte structures and physiology among animal species cause difficulties in formulating a consensus on freezing techniques for all species. This is not the case for humans, as a range of devices and reagents is mostly commercially available, and comparative studies between laboratories can be performed much more easily than for animals. A search for new technologies is also required to identify the mechanisms of cryoinjury by using novel molecular tools such as gene sequencing and proteomic analysis. Several technologies are foreseen to be used in the future, such as microfluidics [163,164,165]. Future prospective issues of fertility preservation can also be examined via the development of cryopreservation/transplantation technologies for oocytes growing in ovarian tissue [161], and also for genetic modifications using novel genome editing tools. Material sciences using three-dimensional (3D) structures (e.g., tissue decellularization scaffolds or fibrin/thrombin structures) can be used to protect oocytes against cryoinjury and promote their development post cryopreservation. Lastly, cross contamination with pathogens in liquid nitrogen is also important for safety reasons, especially during the coronavirus 2019 (COVID-19) pandemic. The presence of angiotensin-converting enzyme (ACE 1-7) has been identified in human ovarian and granulosa cells [166,167]. Thus, there is a major concern on the risk of SARS-CoV-2 contamination in ART procedures, including oocyte retrieval and cryopreservation [166]. Although viral RNA was not detectable in the oocytes and follicular fluid of SARS-CoV-2-positive patients [168,169], larger case studies in all IVF procedures with careful interpretation should be elucidated [169]. For this hygiene security reason, it would be preferable to develop different types of cryodevices using closed systems, such as the High-Security Vitrification™ cryopreservation system [170] in order to avoid cross contamination from liquid nitrogen.

## Data Availability

Not applicable.
